# Caspase-1 inhibition mitigates neonatal hyperoxia-induced vascular and cardiopulmonary inflammation in neonatal rats

**DOI:** 10.1042/CS20242275

**Published:** 2024-12-03

**Authors:** Astrid H. León Silva, Runxia Tian, Sydne Ballengee, Aden Jamal, Swathi Menon, Shreeya V. Chalikonda, Roberta M. Lassance-Soares, April Tan, Joanne Duara, Augusto Schmidt, Karen Young, Shu Wu, Noel Ziebarth, Merline Benny

**Affiliations:** 1 Department of Pediatrics, Batchelor Children’s Research Institute, University of Miami Miller School of Medicine, Florida, U.S.A.; 2 Batchelor Children’s Research Institute, University of Miami Miller School of Medicine, Florida, U.S.A.; 3 DeWitt Daughtry Family Department of Surgery, University of Miami Miller School of Medicine, Florida, U.S.A.; 4 Department of Biomedical Engineering, University of Miami College of Engineering, Florida, U.S.A.

**Keywords:** caspase-1 Inhibition, neonatal hyperoxia, neonatal vascular and cardiopulmonary inflammation

## Abstract

There is a fundamental knowledge gap regarding the effects of neonatal hyperoxia exposure on the systemic vasculature and its repercussions on the cardiopulmonary system. Neonatal hyperoxia exposure induces a pro-inflammatory profile. However, the role of inflammation in the developing vascular tree and cardiopulmonary system is poorly understood. Caspase-1 mediates activation of inflammatory cytokines (IL-1β and IL-18) and gasdermin D (GSDMD), causing pyroptosis and inflammation. We hypothesized that caspase-1 is a critical contributor in neonatal hyperoxia-induced systemic vascular and cardiopulmonary inflammation and that caspase-1 inhibition attenuates hyperoxia-induced vascular stiffness, cardiopulmonary inflammation, and bronchopulmonary dysplasia (BPD) phenotype in a neonatal rat model. Newborn rats randomized to room air (RA) or hyperoxia (85% O_2_) from postnatal day (P) 1 to 14 received caspase-1 inhibitor, VX-765, or placebo. Hyperoxia-exposed pups had increased cardiovascular inflammation and fibrosis, aortic stiffness, pulmonary vascular rarefaction and remodeling, alveolar simplification, and right ventricular hypertrophy. Administration of a caspase-1 inhibitor decreased IL-1β and GSDMD gene and protein expression in the aorta and left ventricle. This was accompanied by reduced aortic stiffness and cardiac fibrosis, improved alveolar structure, pulmonary vascular density and vascular remodeling, and attenuation of right ventricular hypertrophy. Together, our findings suggest that inhibition of the caspase-1 pathway leads to decreased cardiopulmonary inflammation and remodeling. In conclusion, targeting caspase-1 signaling may be a therapeutic strategy to prevent the consequences of vascular and cardiopulmonary inflammation associated with preterm birth and oxygen therapy.

## Introduction

Preterm birth affects 13 million infants globally each year [1]. Advances in neonatal intensive care have improved the survival of preterm infants, and consequently, the prevalence of cardiopulmonary diseases associated with prematurity has increased [2]. Infants born preterm are often exposed to supplemental oxygen (O_2_) and have decreased antioxidant defenses [3,4]. Premature infants frequently develop respiratory distress due to immaturity of the lungs, rendering them highly susceptible to long-term sequelae. Human and animal studies have shown that neonatal hyperoxia induces oxidative stress, which contributes to well-known complications of prematurity, such as bronchopulmonary dysplasia [5]. While much of the research on the effect of prematurity and supplemental oxygen has focused on the subsequent lung injury, its repercussions on the cardiovascular system are less explored. Infants born preterm have an immature cardiovascular system that is not designed for the hemodynamic changes that happen shortly after birth [6]. The combination of these variables leads to oxidative damage, triggering endothelial injury and vascular dysfunction, impairing cardiac remodeling, and even leading to heart failure in early adulthood [7 9]. Emerging evidence shows that prematurity and early life exposures contribute to the developmental programming of cardiopulmonary morbidities in these infants, leading to increased risk for hypertension, vascular stiffness, atherosclerosis, and ischemic heart disease into adulthood [10]. However, the underlying mechanisms leading to cardiovascular dysfunction remain unknown. The need to understand its pathophysiology and to develop strategies to prevent cardiovascular morbidities continues to rise as the population of preterm survivors grows. Despite significant strides in neonatal care, there is no effective therapy for it, underscoring the need for further research in this field.

Most preterm births are secondary to materno-fetal inflammation leading to preterm labor or preterm premature rupture of membranes [11]. Premature infants, due to their immature organ systems, are extremely vulnerable. Postnatally, they are often exposed to a combination of external (hypoxia or hyperoxia, microorganisms, drugs, artificial devices) [12] and epigenetic factors [13], triggering inflammation. Inflammation is a key feature in the pathogenesis of many cardiovascular diseases in adults, including hypertension, acute coronary syndromes, and atherosclerosis [14]. An important concept involved in the mechanism of inflammation is the inflammasome, which has garnered attention as it propagates inflammatory responses [15]. This intracellular multiprotein complex comprised of nucleotide-binding domain, leucine-rich containing receptor family pyrin (NLRP) regulates the activation of caspase-1 triggering the cleavage of proinflammatory cytokines (IL-1β and IL-18) and activation of gasdermin D (GSDMD). This leads to pore assembly, allowing the release of proinflammatory cytokines and the influx of calcium causing pyroptosis (a form of programmed inflammatory cell death) [16]. Pyroptosis can reduce angiogenesis, damage vascular endothelial cells, and cause myocardial fibrosis [17]. Emerging research has highlighted the pivotal role of the inflammasome-caspase-1 axis in the pathogenesis of bronchopulmonary dysplasia (BPD) [18]. Our previous studies have shown that hyperoxia exposure in neonatal mice activates the inflammasome pathway, and caspase-1 inhibition improves lung and brain injury [19]. However, the evidence connecting neonatal hyperoxia, systemic vascular inflammation, vascular stiffness, and GSDMD-mediated pyroptosis is limited.

Studies by our group and others looking at vascular changes in the long term have documented increased aortic stiffness, collagen deposition, and cardiopulmonary dysfunction after neonatal hyperoxia exposure in rodents [20 23]. To our knowledge, no prior study has explored the impact of inflammation in the systemic vasculature and heart inflammation due to neonatal hyperoxia exposure, which could help guide the development of targeted therapies for preterm infants to reduce the burden of cardiovascular after preterm birth [21]. The mechanistic role of inflammasome-caspase-1 signaling in the pathogenesis of hyperoxia-induced vascular stiffness remains unclear. Moreover, whether inhibition of inflammasome-caspase-1 signaling reduces systemic vascular stiffness and cardiovascular fibrosis was heretofore unknown.

We hypothesized that neonatal hyperoxia exposure induces cardiovascular inflammation, systemic vascular stiffness, and cardiopulmonary damage, and moreover, that caspase-1 inhibition is a therapeutic strategy to prevent cardiovascular inflammation, fibrosis, and BPD. The objectives of this study were to investigate whether neonatal hyperoxia exposure alters caspase-1 and GSDMD expression in the myocardium and systemic vasculature and to determine whether caspase-1 inhibition ameliorates neonatal hyperoxia-induced cardiovascular inflammation and fibrosis, aortic stiffness, and BPD phenotype. Our study has important implications, as currently, there are no therapeutic strategies to prevent cardiovascular and pulmonary consequences of preterm birth.

## Materials and methods

### Experimental model

The protocol was approved by the Animal Care and Use Committee at the University of Miami Miller School of Medicine. Pregnant Sprague-Dawley rats were obtained from Charles River Laboratories (Wilmington, MA) and housed with food and water available *ad libitum* at constant temperature (25°C) under 12:12 light/dark cycle. After delivery, rat pups were randomly assigned to normoxia (RA; 21% Oxygen) or hyperoxia (HYP; 85% Oxygen) and to receive daily intraperitoneal (IP) injection of VX-765, 50 mg/kg (Cat#S2228 Selleck Chemical LLC, Houston, TX), or placebo (Cat# D8418 Dimethyl sulfoxide, Sigma-Aldrich, St. Louis, MI) from postnatal day 1 to 14. The pups were housed in a plexiglass chamber with continuous monitoring and oxygen exposure. Mothers were exchanged between RA and Oxygen chamber every 48 h, that was briefly interrupted for animal care (<10 min/day). Litter size was adjusted to 10–12 pups to control for the effect of litter size on growth and nutrition. Both male and female rats were studied at P14.

### *Ex vivo* intrinsic aorta stiffness-atomic force microscopy

Elasticity testing on the descending aorta samples was conducted using a custom-built atomic force microscopy (AFM) system. The aorta samples were adhered to a Petri dish (35 mm, Falcon, 351008) using Liquid Bandage (New Skin, Bridgewater, NJ). The AFM cantilever (5 μm diameter borosilicate glass particle, silicon nitride cantilever, 0.12 N/m, Novascan Technologies, Ames, IA) was lowered onto the samples using a piezoelectric mechanism (60 µm maximal expansion, P-841.40, Physik Instrumente, Germany). The contact interaction between the cantilever and the sample was recorded using a custom code based in IGOR Pro (WaveMetrics, Lake Oswego, OR). The recorded cantilever deflection-indentation curves were used to derive the sample’s force–indentation curves, after factoring out the cantilever deflection on a hard surface and incorporating the spring constant. According to the Hertz contact mechanical model, the force–indentation relation is a function of the effective Young’s modulus of elasticity:


$$F=\frac{4E\sqrt{R}}{3\left(1-v^{2}\right)}D^{3/2}$$

where F is the measured force, E is Young’s modulus, ν is Poisson’s ratio (assumed to be 0.49), R is the radius of the spherical indenter, and D is the measured indentation. A custom curve-fitting MATLAB program was used to analyze the force indentation curves with the Hertz model for spherical indenters [24]. Multiple measurements were taken along the length of the aortic tissue. Raw data were analyzed using the interquartile range method to identify and exclude outliers. Using this method, any value 1.5 times above the third quartile or 1.5 times below the first quartile was excluded as outliers. Since at least 10 repeat measures were acquired per measurement location, we applied this outlier analysis method to the repeat measures for each location. If a point was determined to be an outlier in accordance with the interquartile range method, it was excluded from the average value for that location.

### Western blot

Expression of caspase-1, GSDMD, and TGF-β1 in aortic and heart tissues was assessed by western blot as previously described [20,23]. Briefly, total protein was extracted from frozen aortic and heart tissues using a RIPA buffer as per manufacturer’s protocol. The protein concentrations were measured by BCA protein assay using a commercial kit from Pierce Biotechnology Inc. Western blot was performed as previously described [25]. Total protein (10 μg/sample for aorta and 30 μg/sample for LV) was fractionated by SDS-PAGE on 4%–12% Tris-glycine precast gradient gels (Invitrogen) and then transferred to nitrocellulose membranes (Amersham). The membranes were blocked with 5% non-fat milk at room temperature for 1 hour and then incubated overnight at 4°C with a primary antibody for pro-caspase-1 and cleaved caspase-1 (Mouse Monoclonal antibody, 1:500, Cat# NB100-56565, Bio-Techne, Minneapolis, MN), GSDMD (Rabbit polyclonal antibody, 1:1000, Cat# 39754, Cell Signaling, Danvers, MA), IL-1β (Rabbit monoclonal antibody, 1:500, Cat# ab254360, Abcam, Waltham, MA), TGF-β1 (Rabbit Polyclonal antibody, 1:2000, Cat# ab179695, Abcam, Waltham, MA) and then incubated for 1 h at room temperature with HRP-conjugated secondary antibody. Antibody-bound protein was detected using ECL chemiluminescence methodology (Bio-Rad Cat# 1705061, Hercules, CA). Membranes were then stripped with stripping buffer (Cat# N552-500ml, VWR, Radnor, PA) and re-incubated with an anti-β-actin antibody (monoclonal antibody, 1:10,000; A5441, Sigma Aldrich, Burlington, MA). The protein expression was analyzed by a Quantity One Imaging Analysis Program (Bio-Rad) and normalized by β-actin, a housekeeping protein.

### Real-time PCR

The mRNA expression levels of GSDMD, IL-1β, LOX, and TGF-β1 from aortic and heart tissues were assessed by real-time qPCR as previously described [20,23]. Briefly, total RNA was extracted from frozen aortic and heart tissues using the RNeasy Universal Mini Kit (Cat#217004; Qiagen Inc, Valencia, CA) according to the manufacturer’s instructions. Total RNAs isolated were then reverse transcribed (Cat# A5000; GoScript Reverse Transcription System, Promega, Madison, WI). Real-time qRT-PCR using gene-specific primers and TaqMan Fast Advanced Master Mix (Cat #4444554, Applied Biosystems, Foster City, CA) was performed on an ABI Fast 7500 system (Applied Biosystems) as previously described [26]. The relative mRNA expression of GSDMD (Rn01502567_g1 Invitrogen), LOX (Rn01491829_m1, Invitrogen), TGF-β1 (Rn00572010_m1, Invitrogen) genes was normalized to GAPDH expression, (Cat#Rn99999916_s1, ThermoFisher). In addition, Rat IL-1β (F: GCACAGTTCCCCAACTGGTA, R: ACACGGGTTCCATGGTGAAG), human IL-1β (F: AAGTTCTTCGGTTTGCCGGA, R: TTGTCTCCGTGATCTCCCCT), human GSDMD (F: GATCCGGGACAGGCTGCTA, R: GAGTGGTGCTCGCCATATCA), and human GAPDH (F: CATGACAACTTTGGTATCGTGG, R: CCTGCTTCACCACCTTCTTG) were used.

### Localization of GSDMD and IL-1β in the aorta and heart

GSDMD localization in aortic and heart sections was evaluated by immunohistochemistry. Briefly, aorta and heart tissue sections were incubated with GSDMD Rabbit polyclonal antibody (1:50; PA5-116815, Thermofisher, Waltham, MA) overnight at 4°C. Similarly, the aorta and heart sections were incubated with IL-1β antibody (1:50; ab-283818, Abcam, Waltham, MA). The following day, sections were incubated with biotinylated horseradish peroxidase (HRP)–conjugate goat anti-rabbit secondary antibody (BA-1000, 1:200; Vector Laboratories Inc., Newark, CA) for 1 h, followed by incubation for 45 min with the streptavidin-HRP (Cat# PK-6100; Vector Laboratories Inc.). Peroxidase activity was detected with 3, 3′-Diaminobenzidine (Cat# SK-4100; Vector Laboratories Inc.). Sections were counterstained with hematoxylin, dehydrated, and mounted with Richard-Allan cytoseal XYL (# 8312–4, ThermoFisher Scientific, Waltham, MA).

### Masson’s Trichrome staining

Paraffin-embedded sections of aortic and heart tissue from 2 weeks rats were deparaffinized, rehydrated, and stained with Masson’s Trichrome as per manufacturer’s instructions (Cat #K037; Poly Scientific R&D Corp, Bay Shore, NY) to assess collagen deposition, a marker of fibrosis. Sections were then examined under a light microscope (Zeiss, Germany) and fibrosis was qualitatively determined. Fibrosis was measured as a positively stained area with Masson’s trichrome and was expressed as a percentage of the total area using Image J software. Densitometric quantitative analysis using Image J software (National Institute of Health) [20] was obtained in both aortic and heart tissues. Identical settings and exposure times were used in order to validate comparative analysis between the four groups.

### Immunohistochemistry

α-Smooth muscle actin (α-SMA) localization in heart sections was evaluated by immunohistochemistry [27]. Briefly, heart sections were incubated with α-SMA (Cat# A2547 1:200, Sigma, St Louis, MO) overnight at 4°C. The following day, sections were incubated with biotinylated horse anti-mouse IgG secondary antibody (Cat# BA-2000, 1:400; Vector Laboratories Inc., Burlingame, CA, St. Louis, MO) for 1 h. The sections were then incubated for 45 min with the streptavidin-HRP (Vector Laboratories Inc.). Peroxidase activity was detected with 3, 3′-Diaminobenzidine (Vector Laboratories Inc.). Sections were counterstained with hematoxylin, dehydrated, and mounted with Richard-Allan cytoseal XYL (# 8312–4, ThermoFisher Scientific Waltham, MA). Quantification of immunohistochemistry for α-SMA positive staining was analyzed by calculating the integration optical density value of positive staining using Image J.

### Lung morphometric analysis

Lungs were inflated and perfused with 4% paraformaldehyde (PFA) via a tracheal catheter at a pressure of 15 for 5 min and were left in PFA for 24 h. The following day, the lungs were dehydrated with ethanol and then embedded in paraffin. 5 μm thick sections of paraffin-embedded lung were stained with hematoxylin and eosin. Images of nonoverlapping parenchymal field, with exclusion of vessels and artifacts, were acquired from lungs section of each animal using a magnification of 10× and 20×. The images were analyzed by obtaining mean linear intercept (MLI) and the radial alveolar count (RAC) by a blinded observer as previously described [28].

### Lung angiogenesis

Lung sections were stained with a marker of endothelial cells, polyclonal rabbit antihuman Von Willebrand Factor, vWF (Cat# A0082 1:30; Dako, Carpinteria, CA) and 4ʹ6-di-amidino-2-phenylindole, DAPI (Cat# H-1500 Vector Laboratories, Newark, CA). Non-overlapping parenchymal fields from lung sections of each animal were evaluated. The number of blood vessels (20–50 μm in diameter) was counted in each HPF [29] by a blinded observer.

### Pulmonary vascular remodeling

Lung sections were stained with polyclonal rabbit antihuman vWF (Cat# A0082 1:30; Dako, Carpinteria, CA) and mouse anti-smooth muscle actin, α-SMA (Cat# A2547 1:100, Sigma, St Louis, MO). Non-overlapping parenchymal fields from the lung section of each animal were evaluated. A blinded observer counted the number of blood vessels (20–50 μm in diameter) in each HPF and assessed the degree of muscularization. Medial wall thickness of randomly selected arterioles (20–50 μm in diameter) at 20× was measured [30]. The medial layer of the arteriole thickness was calculated using the formula: 2 x medial layer thickness/average diameter of the vessel × 100%

### Culture and treatment of human umbilical arterial endothelial cell and human aortic smooth muscle cell

Human Umbilical Artery Endothelial Cells, HUAECs (Cat# 202–05n, Cell Applications, San Diego, CA) were cultured in a human MesoEndo growth medium (Cat# 212–500, Cell Applications, San Diego, CA). Human aortic smooth muscle cells (HASMCs) (Cat# 354–05a; Cell Applications, San Diego, CA) were cultured in Smooth Muscle Cell Basal Media. Cultured cells were serum-deprived for 24 h, incubated with a placebo (0.25% DMSO/PBS) or VX765 (50 mM), and exposed to normoxia (21% O_2_, 5% CO_2_) or hyperoxia (95% O_2_, 5% CO_2_) conditions for 48 h. Both gene and protein expression of GSDMD and IL-1β was assessed.

### Multiplex protein quantification

Flash frozen aorta tissues were homogenized in RIPA buffer (Cat# sc-24948, Santa Cruz Biotechnology, Santa Cruz, CA) and centrifuged at 15,000 rpm for 15 min at 4°C. The protein concentration of the supernatant was measured by BCA protein assay (Cat# PI2322 ThermoFisher Scientific, Waltham, MA). All the samples were then diluted for target protein concentration of 100 mg/ml. These protein samples were outsourced to Eve Technologies (Calgary, AB, Canada) for IL-18 protein concentration using the Rat 27-Plex Discovery Assay, a multiplex immunoassay [22]. IL-18 concentration was expressed as picograms/mg of total protein.

### Statistics

For all experiments, n refers to the number of individual rats or individual culture plates. All data are expressed as mean ± standard deviation (SD). *P-*values were calculated using two-way ANOVA with Tukey’s multiple comparisons test (four-group comparison). All analyses were performed using commercially available statistical software packages (GraphPad Prism version 8.3 for Windows, GraphPad Software, San Diego, CA).

## Results

### Neonatal rats exposed to hyperoxia demonstrate activation of the inflammasome pathway in aortic and left ventricular tissue

**Figure 1:**
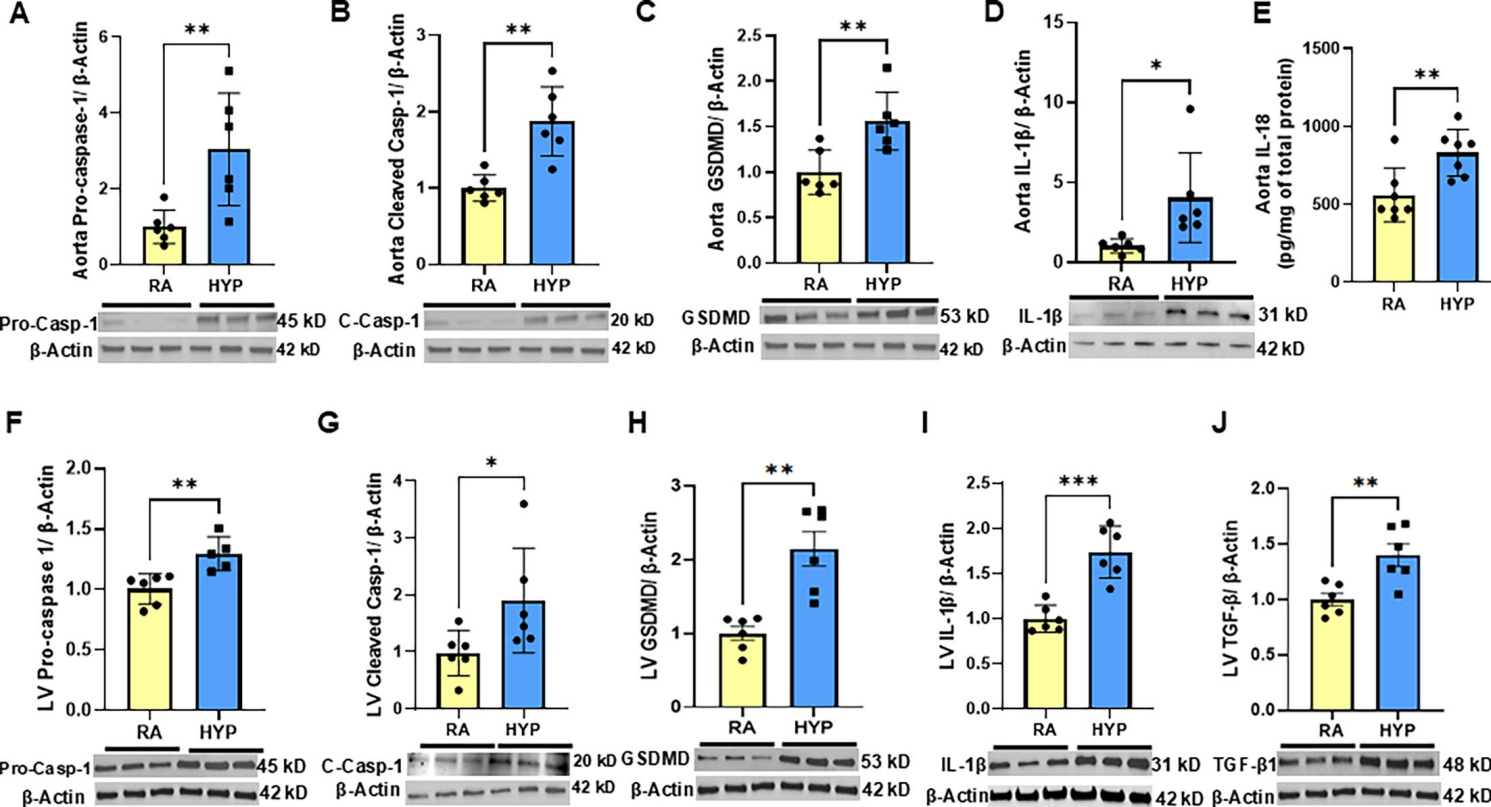
Neonatal rats exposed to hyperoxia demonstrate activation of the inflammasome pathway in aortic and left ventricular tissue. Neonatal hyperoxia increased protein expression of (**A**) Pro-Caspase-1, (**B**) Cleaved-Caspase-1, (**C**) GSDMD, and (**D**) IL-1β in the aorta. Representative Western Blots are shown in the lower panel. (**E**) Multiplex protein analysis of aorta lysate showed increased IL-18 in the aortas of neonatal hyperoxia exposed group. In addition, neonatal hyperoxia exposure increased protein expression of (**F**) Pro-caspase-1, (**G**) Cleaved-Caspase-1, (**H**) GSDMD, (**I**) TGF-β, (**J**) IL-1β in the left ventricle. Representative western blots are shown in the lower panel. *n* = 5–7/group, data are mean ± SD, two tailed *t*-test. **P<0.01; HYP, hyperoxia; RA, room air.

Inflammation is a key contributor to cardiovascular diseases [14]. We first examined the effects of neonatal hyperoxia on key components of the inflammasome pathway, caspase-1, GSDMD, and IL-1β expression in the aorta and heart. When compared with room air exposed pups, neonatal hyperoxia was associated with increased aortic protein expression of pro-caspase-1 (*P*=0.009), cleaved-caspase-1 (*P*=0.001), GSDMD (*P*=0.006), IL-1β (*P*=0.02), and IL-18 (*P*=0.008), key components of the inflammasome pathway (Figure 1A–E). Similarly, neonatal hyperoxia was associated with increased left ventricular protein expression of pro-caspase-1 (*P*=0.005), cleaved-caspase-1 (*P*=0.04), GSDMD (*P*=0.001), IL-1β (*P*=0.0002), and TGF-β (*P*=0.001) (Figure 1F–J), suggesting increased inflammation in the vascular bed and in the heart.

### Caspase-1 inhibition decreases systemic vascular inflammation in neonatal rats exposed to hyperoxia

**Figure 2:**
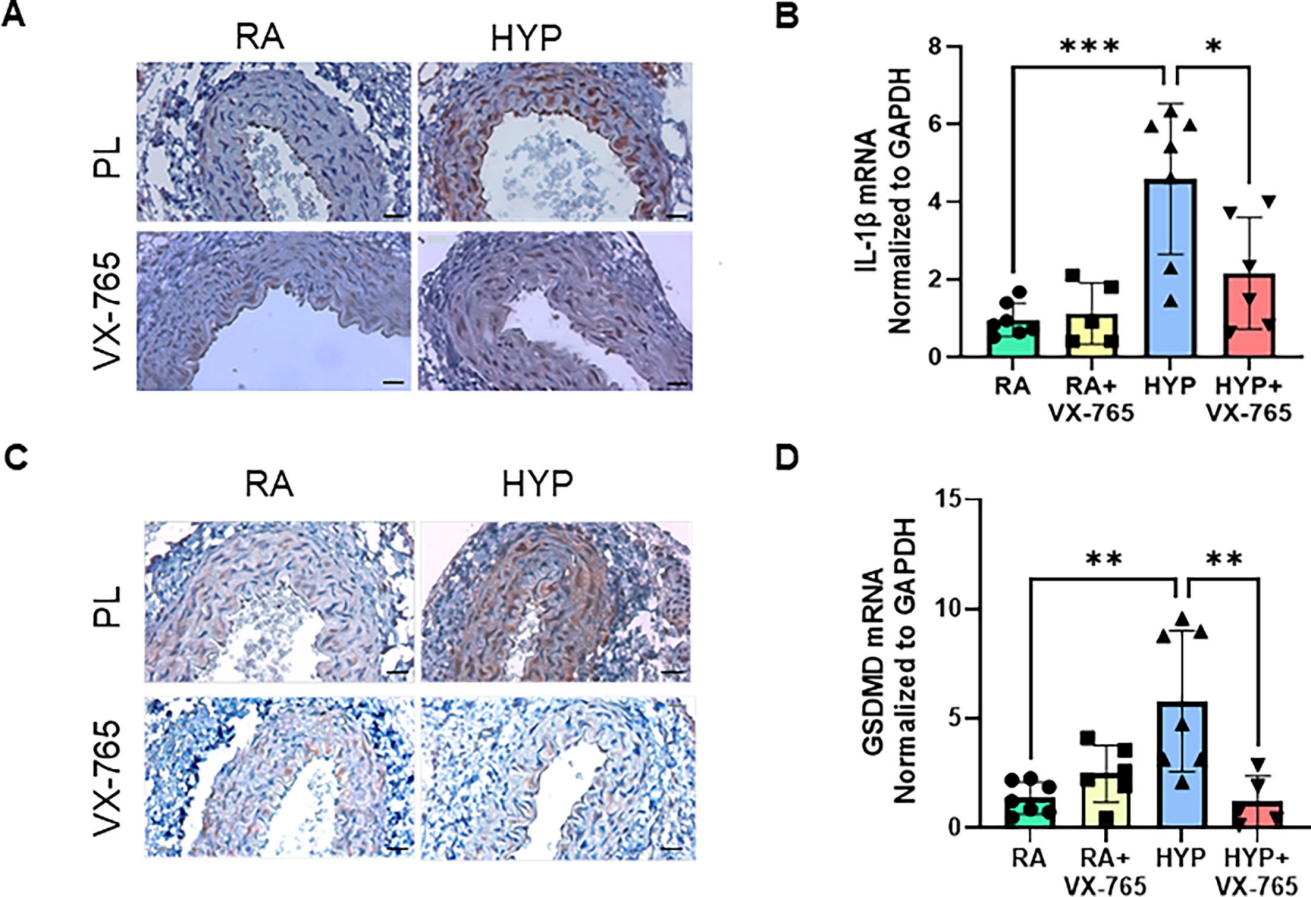
Caspase-1 inhibition decreases aortic tissue IL-1β and GSDMD expression in neonatal rats exposed to hyperoxia. (**A**) Representative images of IL-1β immunostaining (brown staining) in aortic sections. (**B**) Treatment with VX-765 in hyperoxia-exposed neonatal pups decreased IL-1β gene expression in aortic tissue. (**C**) Representative images of GSDMD immunostaining (brown staining) in aortic sections. (**D**) Treatment with VX-765 in hyperoxia-exposed neonatal pups decreased GSDMD gene expression in aortic tissue. Scale bars = 50 μm. *n* = 5–8/group, data are mean ± SD, two-way ANOVA. *P<0.05, **P<0.01, ***P<0.001; HYP, hyperoxia; PL, placebo; RA, room air; VX-765, caspase-1 Inhibitor.

Caspase-1 plays an important role in vascular inflammation and is critical in modulating key components of the inflammasome pathway, IL-1β and GSDMD [31]. Hence, we next probed the contributory role of caspase-1 inhibition in hyperoxia-induced vascular inflammation. Immunostaining to determine the aortic spatial expression of IL-1β and GSDMD demonstrated that both IL-1β and GSDMD were highly expressed in the tunica intima and tunica media of the aorta following exposure to hyperoxia. Administration of a caspase-1 inhibitor decreased the expression of IL-1β and GSDMD in the aorta (Figure 2A and C), suggesting reduced vascular inflammation. In addition, we showed that newborn hyperoxia-placebo-treated rats, when compared with the normoxia placebo-exposed group, had increased gene expression of IL-1β (*P*<0.001) and GSDMD (*P*<0.01) in aortic tissues. Administration of a caspase-1 inhibitor (VX-765) during the neonatal period significantly decreased IL-1β and GSDMD mRNA levels, suggesting that caspase-1 inhibition decreases vascular inflammation (Figure 2B and D).

### Caspase-1 inhibition blunts cardiac inflammation in neonatal rats exposed to hyperoxia

**Figure 3:**
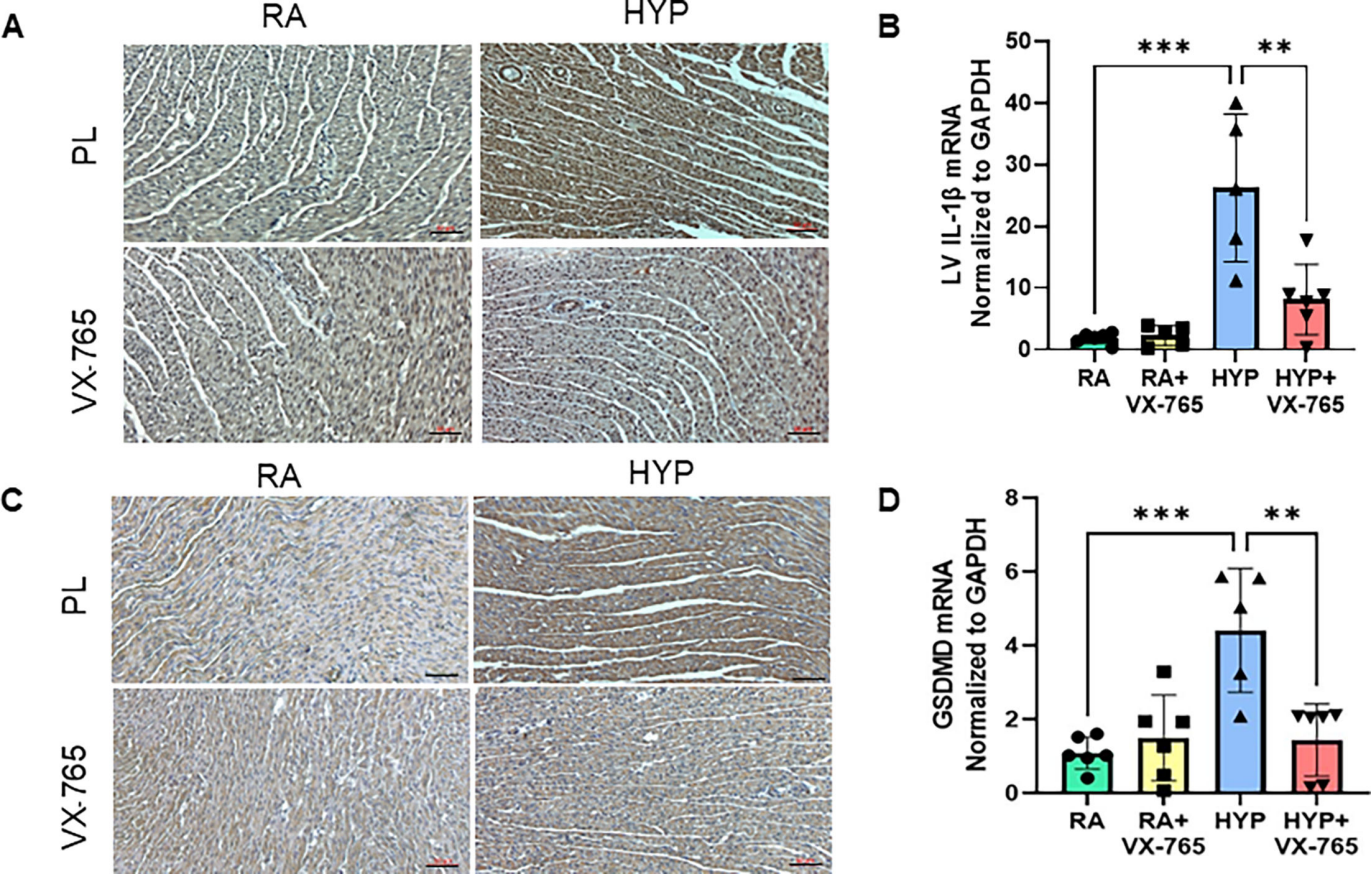
Caspase-1 inhibition decreases left ventricular (LV) IL-1β and GSDMD expression in neonatal rats exposed to hyperoxia. (**A**) Representative images of IL-1β immunostaining (brown staining) in LV sections sampled at postnatal day 14. (**B**) Treatment with VX-765 in hyperoxia-exposed neonatal pups decreased IL-1β gene expression in LV. (**C**) Representative images of GSDMD immunostaining (brown staining) in LV sections. (**D**) Treatment with VX-765 in hyperoxia-exposed neonatal pups decreased GSDMD gene expression in LV. Scale bars = 50 μm. *n* = 5–6/group, data are mean ± SD, two-way ANOVA. **P < 0.01, ***P < 0.001; HYP, hyperoxia; PL, placebo; RA, room air; VX-765, caspase-1 Inhibitor.

In the next step, we sought to dissect the contributory role of caspase-1, IL-1β, and GSDMD in cardiac inflammation [32]. Immunostaining demonstrated that whereas IL-1β and GSDMD were highly expressed in the left ventricle after exposure to hyperoxia, administration of a caspase-1 inhibitor blunted this response in the hyperoxia-exposed group (Figure 3A and C). In addition, when compared with the normoxia placebo-exposed group, the hyperoxia placebo-exposed rat pups had increased gene expression of IL-1β (*P*<0.001) and GSDMD (*P*<0.001) in their left ventricular tissue. These levels were significantly decreased in hyperoxia-exposed rat pups treated with the caspase-1 inhibitor (Figure 3B and D).

### Caspase-1 inhibition ameliorates vascular stiffness and lysyl oxidase (LOX) gene expression in neonatal rats exposed to hyperoxia

**Figure 4:**
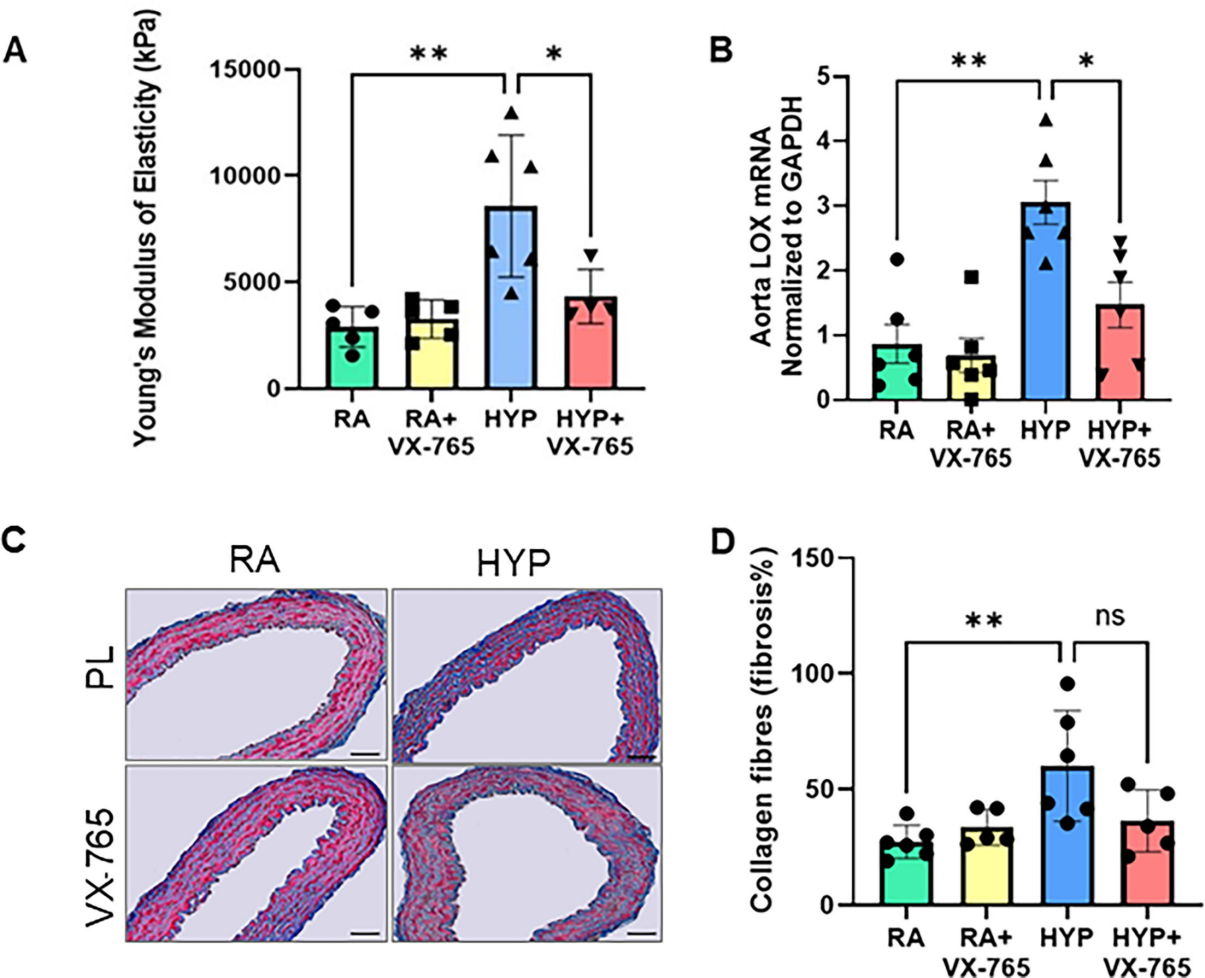
Caspase-1 inhibition decreases aortic vascular stiffness and LOX gene expression in neonatal rats exposed to hyperoxia. (**A**) Treatment with caspase-1 inhibitor decreased vascular stiffness as represented by decreased Young’s modulus of elasticity in hyperoxia-exposed rat pups. (**B**) Capsase-1 inhibition decreased LOX gene expression in the aorta of hyperoxia-exposed treated rat pups. (**C**) Masson’s Trichrome and (**D**) fibrosis quantification in the tunica media of aorta shows that neonatal hyperoxia increased aortic fibrosis, but there was no difference between room air treated and hyperoxia-exposed treated animals. Scale bars = 50 μm. *n* = 4–6/group, data are mean ± SD, two-way ANOVA with Tukey’s multiple comparisons test. *P<0.05, **P<0.01. HYP, hyperoxia; PL, placebo; RA, room air; VX-765, caspase-1 Inhibitor.

Vascular stiffness is related to collagen and extracellular alterations and has been correlated with cardiovascular diseases of different etiologies [33]. Inflammation leads to collagen deposition and fibrosis. Lysyl oxidase (LOX), a family of extracellular matrix (ECM) cross-linking enzymes, plays an important role in fibrogenesis [34]. To assess the degree of vascular stiffness, atomic force microscopy (AFM) was used to calculate Young’s modulus of elasticity in the aorta, where a higher Young’s modulus is directly related to the degree of stiffness. Newborn placebo-treated hyperoxia-exposed rats exhibited increased vascular stiffness in the aorta, as evidenced by increased Young’s modulus. Treatment with the caspase-1 inhibitor VX-765 significantly improved vascular stiffness of the aorta (*P*<0.05) (Figure 4A). In addition, hyperoxia-exposed rat pups had increased levels of LOX gene expression that decreased after administration of a caspase-1 inhibitor (Figure 4B). As fibrosis promotes vascular stiffness, we next assessed histologic markers of fibrosis using Masson’s Trichrome in the aorta. Administration of caspase-1 inhibitor showed no significant difference in aortic fibrosis between normoxia placebo-exposed and normoxia-VX-765 exposed neonatal rats. Whereas hyperoxia-placebo exposed rat pups had increased aortic fibrosis, as evidenced by Masson’s Trichrome staining of the aorta, treatment with caspase-1 inhibitor showed a trend to decrease fibrosis. However, it was not statistically significant (Figure 4C–D).

### Caspase-1 inhibition exerts anti-fibrotic effects in the left ventricle of neonatal rats exposed to hyperoxia

**Figure 5:**
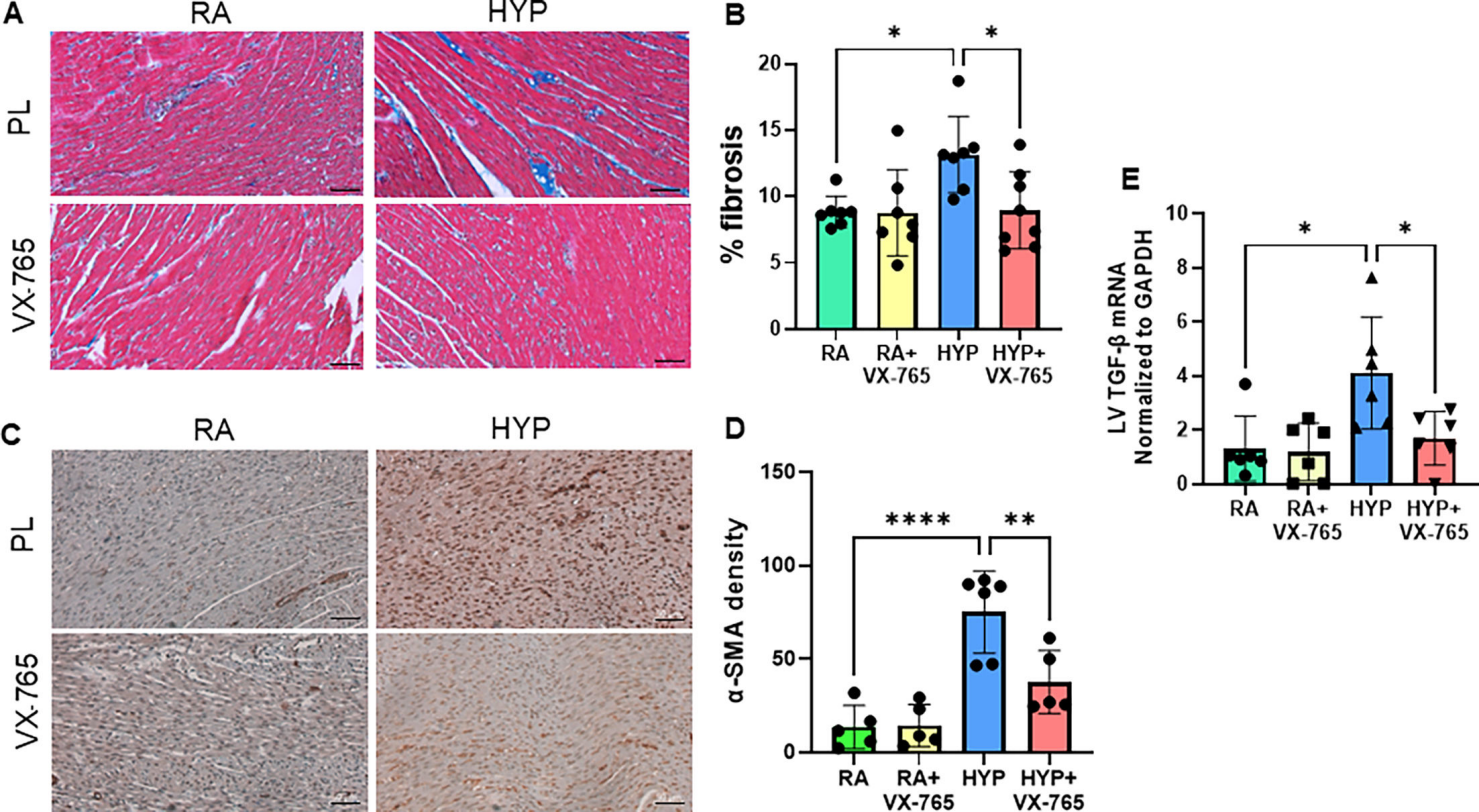
Caspase-1 inhibition exerts antifibrotic effects in the left ventricle (LV) of neonatal rats exposed to hyperoxia. (**A**) Representative images of LV with Masson’s trichrome staining showing decreased collagen (blue) in the hyperoxia-exposed caspase-1 inhibitor treated neonatal pups (**B**) Fibrosis quantification in LV using Image J software. (**C-D**) Immunohistochemical staining of α-SMA and quantification in LV demonstrated that treatment with caspase-1 inhibitor in the hyperoxia-exposed rat pups attenuated α-SMA expression, indicating decreased myocardial fibrosis. (**E**) TGF-β mRNA level demonstrate that treatment with caspase-1 inhibitor attenuated cardiac fibrosis in hyperoxia-exposed neonatal pups. Scale bars = 50 μm. *n* = 6–8/group, data are mean ± SD, two-way ANOVA with Tukey’s multiple comparisons test. *P < 0.05. HYP, hyperoxia; PL, placebo; RA, room air; VX-765, caspase-1 Inhibitor.

Cardiac fibrosis is the result of myofibroblast formation and collagen deposition, often secondary to inflammation [35]. Left ventricular (LV) tissue stained with Masson’s Trichrome showed a significant increase in collagen deposition in LV of placebo-treated rats exposed to hyperoxia (*P*<0.05). However, this increase was significantly reduced in hyperoxia-exposed animals that were treated with the caspase-1 inhibitor (Figure 5A–B). Additionally, cardiac fibrosis was further quantified by immunohistochemistry for α- SMA in the LV. This demonstrated that the hyperoxia-exposed rat hearts exhibited increased myofibroblasts, as evidenced by the increased α-SMA expression. In contrast, treatment with a caspase-1 inhibitor in the hyperoxia-exposed rat pups reduced α-SMA expression, indicating decreased myocardial fibrosis (Figure 5C–D). Moreover, hyperoxia-placebo-treated rat pups had increased gene expression of pro-fibrotic and pro-inflammatory marker, TGF-β1 (*P*<0.05). Treatment with the caspase-1 inhibitor in hyperoxia-exposed rats reduced TGF-β1 levels (Figure 5E). This suggests that caspase-1 inhibition may improve neonatal hyperoxia-induced cardiac fibrosis.

### Caspase-1 inhibition attenuates hyperoxia-induced systemic vascular endothelial and smooth muscle inflammation

**Figure 6:**
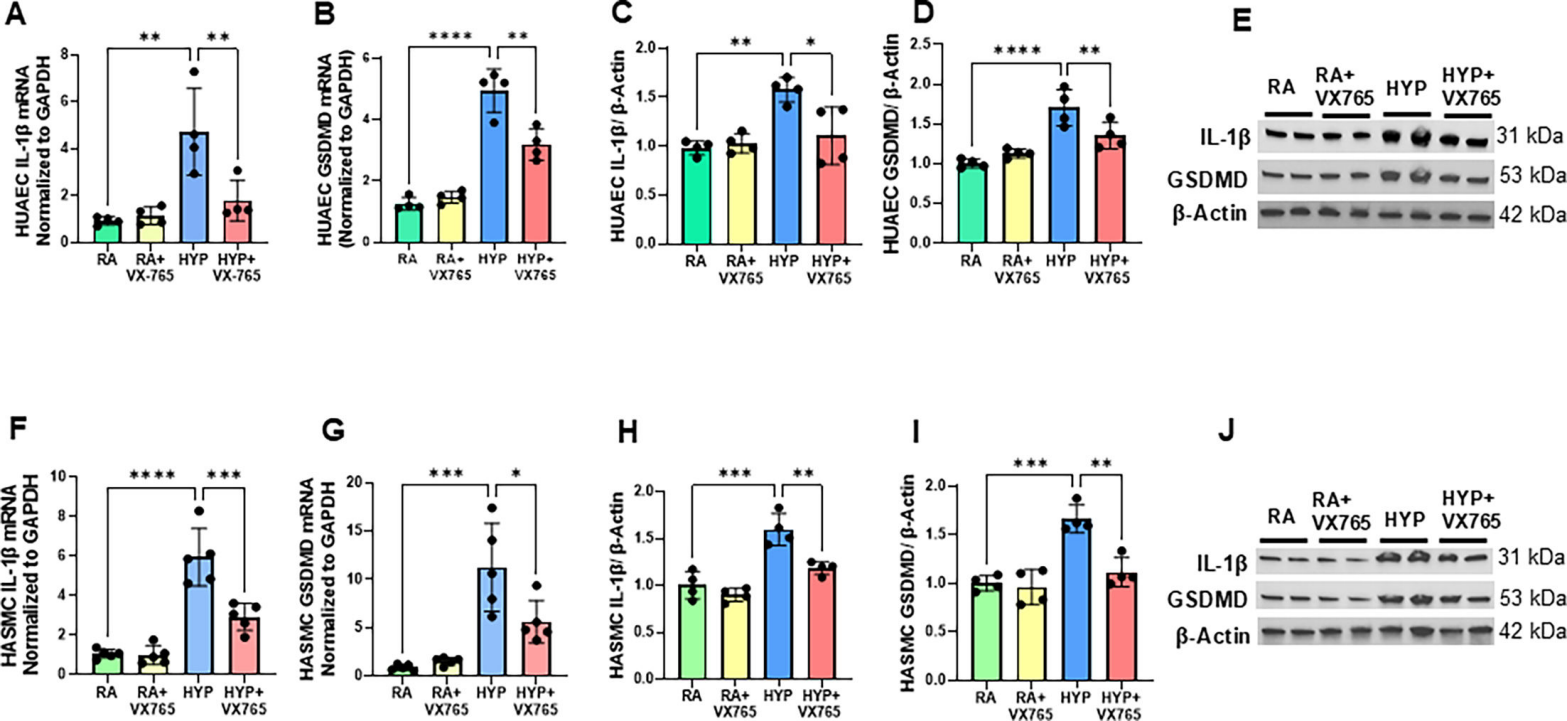
Caspase-1 inhibition attenuates hyperoxia-induced systemic vascular endothelial and smooth muscle inflammation. Treatment with caspase-1 inhibitor decreased gene expression of (**A**) IL-1β, (**B**) GSDMD and protein expression of (**C**) IL-1β, (**D**) GSDMD in hyperoxia-exposed endothelial cells. (**E**) Representative western blots of IL-1β and of GSDMD in the endothelial cells. In addition, caspase-1 inhibitor also reduced gene expression of (**F**) IL-1β (**G**) GSDMD and protein expression of (**H**) IL-1β, (**I**) GSDMD in hyperoxia-exposed smooth muscle cells. (**J**) Representative western blots of IL-1β and of GSDMD in the smooth muscle cells. *n* = 4–5/group, data are mean ± SD, two-way ANOVA with Tukey’s multiple comparisons test. *P<0.05 **P<0.01, ***P<0.001; ****P<0.0001. HYP, hyperoxia; PL, placebo; RA, room air; VX-765, caspase-1 Inhibitor.

Endothelial dysfunction is well-known to predispose to cardiovascular disease [36], and vascular smooth muscle cells compose the majority of the vascular wall and retain phenotypic plasticity in response to various stimuli [37]. To study the role of caspase-1 in hyperoxia-induced systemic vascular dysfunction, we evaluated HUAECs and HASMCs. Of note, the aorta is a direct extension of the umbilical artery in neonates. Whereas hyperoxia exposure increased the gene expression of IL-1β and GSDMD both in the HUAECs (Figure 6A–B) and HASMCs (Figure 6F–G), incubation of these cells with a caspase-1 inhibitor reversed these changes. Similarly, western blot analysis demonstrated that caspase-1 inhibitor reduced the protein expression of IL-1β and GSDMD in both the HUAECs (Figure 6C–E) and HASMCs (Figure 6H–J). This suggests that treatment with a caspase-1 inhibitor may ameliorate the inflammatory response of the hyperoxia-exposed systemic vascular endothelial and smooth muscle cells.

### Caspase-1 inhibition improves lung angiogenesis and vascular remodeling in neonatal rats exposed to hyperoxia

**Figure 7:**
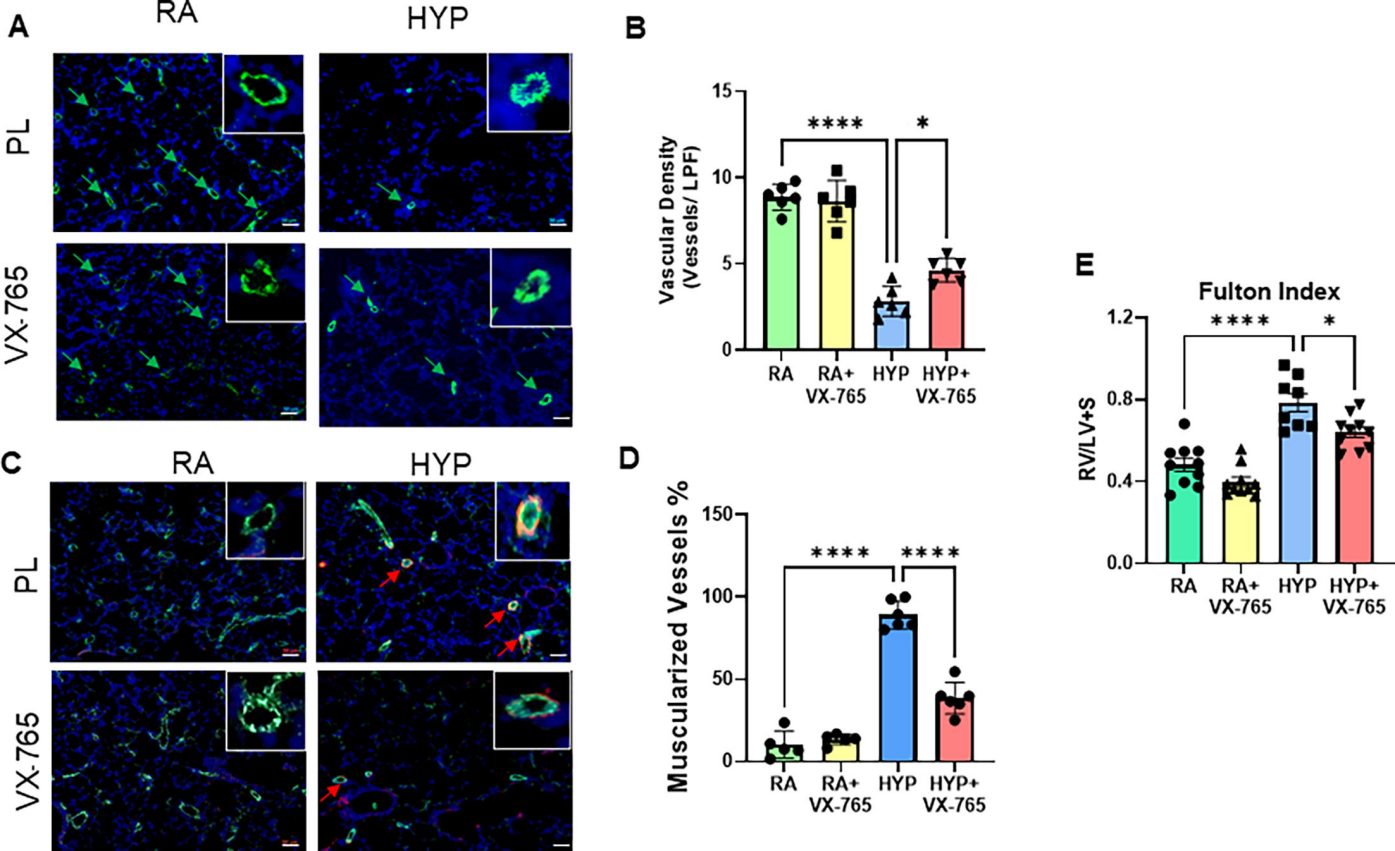
Caspase-1 inhibition improves lung angiogenesis, vascular remodeling, and right ventricular hypertrophy in neonatal rats exposed to hyperoxia. (**A**) Representative lung sections stained with Von Willebrand Factor (vWF-green), 4’6-diamidino-2-phenylindole (DAPI-blue) showing increased (**B**) pulmonary vascular density quantification (green arrows denoting pulmonary vessels) in hyperoxia-exposed caspase-1 inhibitor treated neonatal rats. (**C**) Immunostaining of lung sections with vWF (green), DAPI (blue), and smooth muscle actin (SMA-red) showing decreased (**D**) percentage of muscularized vessels (red arrows denoting muscularized vessels) in hyperoxia-exposed-VX-765 treated group. (**E**) Caspase-1 inhibitor improved right ventricular hypertrophy expressed by Fulton Index in neonatal hyperoxia-exposed rat pups. Original magnification 20X. Scale bars = 50 μm. *n* = 5–10/group, data are mean ± SD, two-way ANOVA with Tukey’s multiple comparisons test. *P<0.05, ****P<0.0001. HYP, hyperoxia; PL = placebo; RA , room air; VX-765, caspase-1 Inhibitor.

Dysfunctional angiogenesis and pulmonary vascular remodeling are key features of BPD. We next evaluated lung angiogenesis by pulmonary vascular density and pulmonary vascular remodeling by the percentage of muscularized vessels. Exposure to hyperoxia significantly reduced pulmonary vascular density and increased the percentage of muscularized vessels in the hyperoxia-placebo-exposed rat pup, as compared with the normoxia placebo-exposed group. Treatment with a caspase-1 inhibitor increased vascular density and reduced the percentage of muscularized vessels in the lungs (Figure 7A–D). Additionally, whereas hyperoxia-exposed placebo-treated animals had increased right ventricular hypertrophy, administration of caspase-1 inhibitor reduced right ventricular hypertrophy (Figure 7E–F).

### Caspase-1 inhibition demonstrates an alveolar protective role in neonatal rats exposed to hyperoxia

**Figure 8:**
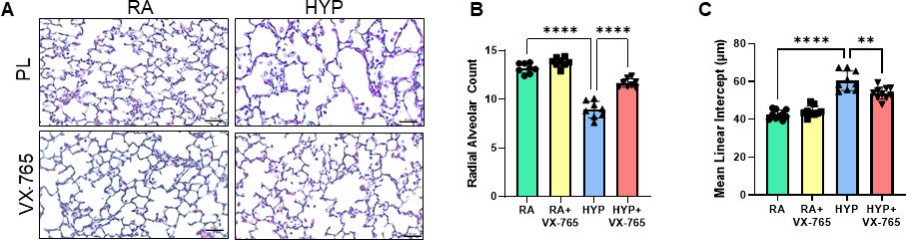
Caspase-1 inhibition improves alveolar development in neonatal rats exposed to hyperoxia. (**A**) Hematoxylin and eosin-stained lung sections demonstrating improved alveolar structure in hyperoxia-exposed rats treated with caspase-1 inhibitor VX-765. Morphometric analysis showed significantly (**B**) increased radial alveolar count (RAC) and (**C**) decreased mean linear intercept (MLI) in hyperoxia VX-765-treated groups. Scale bars = 50 μm. *n* = 8/group, data are mean ± SD, two-way ANOVA with Tukey’s multiple comparisons test. **P<0.01, ****P<0.0001. HYP, hyperoxia; PL, placebo; RA , room air; VX-765, caspase-1 Inhibitor.

BPD is characterized by an arrest of alveolar development. Therefore, we assessed the effects of caspase-1 inhibition on lung alveolarization in this experimental BPD model. Newborn placebo-treated hyperoxia-exposed rats demonstrated severe destruction of the alveolar architecture, as evidenced by enlarged and simplified alveoli (Figure 8A). Lung morphometric analysis demonstrated significantly decreased RAC (Figure 8B) and increased MLI (Figure 8C) in hyperoxia-exposed placebo-treated rats. Administration of caspase-1 inhibitor significantly increased RAC and reduced MLI (Figure 8B–C).

## Discussion

 Mounting clinical evidence shows that preterm-born survivors are at increased risk of cardiopulmonary diseases [38 40], yet little is known about the molecular mechanisms that lead to these cardiopulmonary diseases. Inflammation is one of the crucial pathophysiological causes of cardiovascular dysfunction [41]. This study aimed to delve into the mechanisms behind neonatal hyperoxia-mediated inflammasome activation in vascular stiffness and cardiopulmonary inflammation and explore the role of caspase-1 inhibition in preventing this cardiopulmonary injury. This study demonstrates a significant contributory role for the caspase-1-GSDMD axis in modulating systemic vascular stiffness, cardiopulmonary inflammation, and cardiopulmonary remodeling. Moreover, we demonstrate a novel role for caspase-1 inhibition in preventing neonatal hyperoxia-induced aortic stiffness, cardiac inflammation, and cardiac fibrosis. This was associated with decreased IL-1β and GSDMD levels in both the aorta and heart. Additionally, in the lung, caspase-1 inhibition improved neonatal hyperoxia-induced alveolar simplification, pulmonary vascular rarefaction and remodeling, and right ventricular hypertrophy. Together, these findings suggest that pharmacological caspase-1 inhibition may prevent vascular and cardiopulmonary consequences of preterm birth by decreasing cardiopulmonary inflammation.

One important finding of our study is that hyperoxia exposure concurrently induces inflammation in the systemic vasculature, heart, and lungs of neonatal rat pups by simultaneously activating caspase-1, IL-1β, and GSDMD. This study utilized the neonatal hyperoxia rat model, which emulates the condition of prematurity where antioxidant defenses are compromised, and supplemental oxygen renders the organs vulnerable to oxidative damage. Our findings are consistent with our prior studies, which show that caspase-1 is elevated in the lung and brain of neonatal hyperoxia-exposed mice [42]. Other studies have shown that NLRP3/IL-1β is critically involved in rodent neonatal hyperoxia-induced lung injury [18] and caspase-1 inhibition was shown to inhibit inflammasome assembly and atherosclerosis by alleviating mitochondrial damage and boosting mitophagy using cell cultures and an adolescent murine model exposed to high-fat diet to facilitate the formation of atherosclerotic lesions [43]. An additional study done in adult mice showed that caspase-1 inhibitor alleviates clamping-induced ischemia-reperfusion injury in the lung by suppressing endothelial pyroptosis and barrier dysfunction [44].

To explore the therapeutic potential of targeting caspase-1 in neonatal hyperoxia-induced cardiovascular inflammation, we administered a caspase-1 inhibitor, VX-765. Whereas hyperoxia-exposed rat pups exhibited increased inflammatory markers, IL-1β and GSDMD, in the aorta and heart, administration of a caspase-1 inhibitor reduced their levels. Moreover, caspase-1 inhibitor administration improved vascular stiffness in the hyperoxia-exposed animals, shown as reduced Young’s modulus of elasticity in the aorta. Vascular stiffness represents the earliest manifestations of adverse structural and functional changes within the vascular wall with a reduced capacity for arterial expansion and recoil in response to pressure changes and has been correlated with cardiovascular diseases of different etiologies [45]. Inflammation plays a major role in vascular stiffening, including those caused by endothelial dysfunction, extracellular matrix degradation, oxidative stress, and degradation of collagen [46]. Vascular inflammation and prematurity [47] increase arterial stiffness by enabling vascular fibrosis. Interestingly, caspase-1 inhibition did not completely shield against vascular fibrosis in the hyperoxia-exposed animals. However, caspase-1 inhibition improved LOX gene expression in the aorta of hyperoxia-exposed animals. LOX is a key mediator of collagen cross-linking, and up-regulation of LOX has been associated with vascular stiffness and extracellular matrix remodeling [48]. It is plausible that the reduction in aortic LOX contributed to improvement in vascular stiffness in the hyperoxia-exposed caspase-1 inhibitor-treated rat pups. In keeping with our in vivo findings of vascular inflammation, our in vitro studies using HUAEC and HASMC revealed that caspase-1 inhibition offered protection against vascular inflammation induced by hyperoxia exposure. These results, along with our novel findings that caspase-1 inhibition improves hyperoxia-induced vascular stiffness and vascular inflammation, support our hypothesis that targeting caspase-1 may be a therapeutic strategy for preserving vascular development in the neonates.

Another important finding in our study is that caspase-1 inhibition improves cardiac fibrosis and remodeling in neonatal hyperoxia-exposed rat pups, thus revealing important effects of caspase-1 inhibition on heart plasticity during neonatal life. Fibroblasts are the main producers of collagen in the injured heart, and they undergo dramatic phenotypic transitions during cardiac repair. In the proliferative phase of cardiac repair, the fibroblasts convert to myofibroblasts, expressing contractile proteins such as α-smooth muscle actin (α-SMA) and synthesize large amount of extracellular matrix proteins, including collagen [49,50]. Caspase-1 inhibition blunted the collagen synthesis and in parallel decreased myofibroblast in the hyperoxia-exposed hearts. Few studies have described the developmental programming of cardiovascular diseases under different deleterious developmental exposures. Bertagnolli et al. showed that pups exposed to transient neonatal hyperoxia had increased cardiomyocyte surface area at 10 days of life, and by 4 weeks of life, the increased cardiomyocyte area persisted with associated fibrosis [51,52]. In contrast to prematurity-induced cardiac remodeling, several studies have investigated the role of caspase-1 in adult cardiac disease. Increased expression of caspase-1 has been demonstrated in murine and human adult heart failure [53]. Caspase-1 deficient mice with myocardial infarction have displayed a significant reduction in mortality and reduced cardiomyocyte pro-apoptosis, decreased MMP-3 activity, and IL-18 production [54]. Similarly, in a cardiac hypertrophic preconditioning mouse model of transverse aortic constriction, caspase-1 overexpression abolished the protective effect of hypertrophic preconditioning on fibrosis and inflammation [55]. Zheng et al. proved that the absence of inflammasome and caspase-1 deficiency alleviates atherosclerosis [56]. Additional research indicates that heart failure, myocardial ischemia-reperfusion injury, diabetic cardiomyopathy, and other diseases show myocardial injury due to cardiomyocyte pyroptosis [57]. VX-765 is the prodrug of its active compound VRT-043198, which is a caspase-1 inhibitor that also inhibits caspase-4, another caspase involved in inflammatory responses [58]. Studies have shown that VX-765 can protect the heart against acute ischemia-reperfusion injury (IR) in a rat model [59]. It may exert its protective effects by interacting with the reperfusion injury salvage kinase (RISK) pathway, potentially preventing loss of cardiac glycolytic enzymes, mitochondrial complex 1 activity, and suppressing release of lactate dehydrogenase [59,60]. Thus, an increasing number of experiments, both *in vivo* and *in vitro*, have proven the role of caspase-1 in cardiovascular disease, and a possible new strategy for their treatment is the use of drugs to inhibit pyroptosis [61].

Bujak et al.^62^ summarize current data on the role of IL-1β in cardiovascular pathology, including atherosclerotic disease and regulation of an inflammatory response, which triggers abnormal remodeling after myocardial infarction and promotes myocardial hypertrophy and cardiomyocyte apoptosis. One double-blinded randomized clinical trial implementing a therapeutic monoclonal antibody targeting IL-1β in adults (CANTOS) showed a significantly lower rate of recurrent cardiovascular events when compared with placebo [63]. According to clinicaltrials.gov, two clinical trials have been performed to study the effects of VX-765 in human subjects, one for patients with psoriasis (NCT00205465) and another for treatment-resistant partial epilepsy (NCT01048255) and the results are pending [64]. Further studies in this field are needed to prove the efficacy of these promising therapies.

Inflammation is a key contributor to aberrant lung development and remodeling in preterm infants with BPD. Our present study showed that hyperoxia-exposed rat pups had impaired alveolarization, lung angiogenesis, vascular remodeling, and increased right ventricular hypertrophy. Caspase-1 inhibition attenuated these findings associated with neonatal hyperoxia-induced lung injury in the rat pups. Recent studies in a murine model of BPD with pulmonary hypertension using micro-CT showed rarefaction of small pulmonary vessels and increase in the larger vessels. Infants with BPD develop higher pulmonary vascular resistance, pulmonary hypertension, and RV dysfunction as a result of reduced vascular density, abnormal vasculature architecture, and vasoreactivity [65]. One of the caspase-1 inhibitors, Ac-YVAD-CMK, had been shown to be lung protective in hyperoxia-mediated neonatal lung injury in mice [19]. In line with this, another study has shown that this caspase-1 inhibitor VX-765 suppressed the progression of hypoxia- and monocrotaline-induced pulmonary hypertension [66]. In another model of hypoxia-induced pulmonary hypertension, caspase-1^-/-^ mice showed reduced pulmonary artery muscularization and right ventricular remodeling when compared with wildtype mice [67].

To the best of our knowledge, the current study is the first to demonstrate a protective role for caspase-1 inhibition in preventing neonatal hyperoxia-induced aortic stiffness, cardiac inflammation, and cardiac fibrosis, associated with decreased IL-1β and GSDMD levels in both the aorta and heart. As with any study, there were limitations to the study described here. First, the neonatal hyperoxia model does not fully recapitulate the vascular dysfunction and cardiopulmonary dysfunction seen in preterm infants, as this is multifactorial in etiology and would need to be evaluated in other experimental models. Second, while we show that caspase-1 inhibition improves vascular stiffness and cardiopulmonary remodeling, further mechanistic studies exploring the posttranscriptional effects of caspase-1 inhibition on inflammation and fibrosis would be important.

In summary, this study reports a novel role for caspase-1 in neonatal hyperoxia-induced systemic vascular stiffness and cardiac remodeling in neonatal rat pups. Strategies that target the caspase-1 signaling may improve vascular stiffness and cardiopulmonary dysfunction in preterm-born survivors. Further studies are warranted to assess specific mechanisms by which caspase-1 inhibition is protective in vascular function and cardiovascular inflammation in preterm-born survivors. Targeting the caspase-1 pathway may be a potential strategy to abrogate the vascular and cardiopulmonary consequences of preterm birth.

**Clinical Perspective**

- Emerging evidence shows that prematurity and neonatal hyperoxia exposure contribute to the developmental programming of cardiopulmonary morbidities in premature infants; however, the pathogenic mechanisms are unknown.
- Using a neonatal hyperoxia rodent model, which recapitulates the condition of prematurity, this study demonstrates a novel role for caspase-1 inhibition in preventing neonatal hyperoxia-induced aortic stiffness, cardiovascular inflammation, cardiac fibrosis, and improved alveolar structure, pulmonary vascular density and vascular remodeling, and attenuation of right ventricular hypertrophy.
- Our study has important implications as currently, there are no therapeutic strategies to prevent cardiopulmonary consequences of preterm birth. This study demonstrates a significant contributory role for the caspase-1-GSDMD axis in neonatal hyperoxia-induced cardiopulmonary remodeling and that pharmacological caspase-1 inhibition may prevent vascular and cardiopulmonary consequences of preterm birth.

## Supplementary material

online supplementary material 1.

## Data Availability

The original contributions presented in this study are included in the article; further inquiries can be directed to the corresponding author.

## Open Access

This article has been published open access under our Subscribe to Open programme, made possible through the support of our subscribing institutions, learn more here: https://portlandpress.com/pages/open_access_options_and_prices#conditional

## Abbreviations

AFM: atomic force microscopy
GSDMD: gasdermin D
HRP: horseradish peroxidase
HYP: hyperoxia
