# Supplementary material for: Caspase-1 inhibition mitigates neonatal hyperoxia-induced vascular and cardiopulmonary inflammation in neonatal rats
Source: Clin Sci (Lond). 2024 Dec 3;139(23):1611–27. doi: 10.1042/CS20242275 (PMC12751044; doi:10.1042/CS20242275)
Supplement: online supplementary material 1. [file CS-139-23-CS20242275-s001.docx]

**SUPPLEMENTAL MATERIAL**

**Caspase-1 Inhibition Mitigates Neonatal Hyperoxia-Induced Vascular and Cardiopulmonary Inflammation in Neonatal Rats**

Astrid H. León Silva^1,2^, Runxia Tian^1,2^, Sydne Ballengee^1,2^, Aden Jamal^1,2^, Swathi Menon^1,2^, Shreeya V. Chalikonda, Roberta M. Lassance-Soares^3^, April Tan^1,2^, Joanne Duara^1,2^, Augusto Schmidt^1,2^, Karen Young^1,2^, Shu Wu^1,2^, Noel Ziebarth^4^, Merline Benny^1,2^

^1^Department of Pediatrics, ^2^Batchelor Children’s Research Institute, University of Miami Miller School of Medicine, Florida. ^3^DeWitt Daughtry Family Department of Surgery, University of Miami Miller School of Medicine, Florida. ^4^Department of Biomedical Engineering, University of Miami College of Engineering, Florida.

*Correspondence

E-mail: mxk968@med.miami.edu


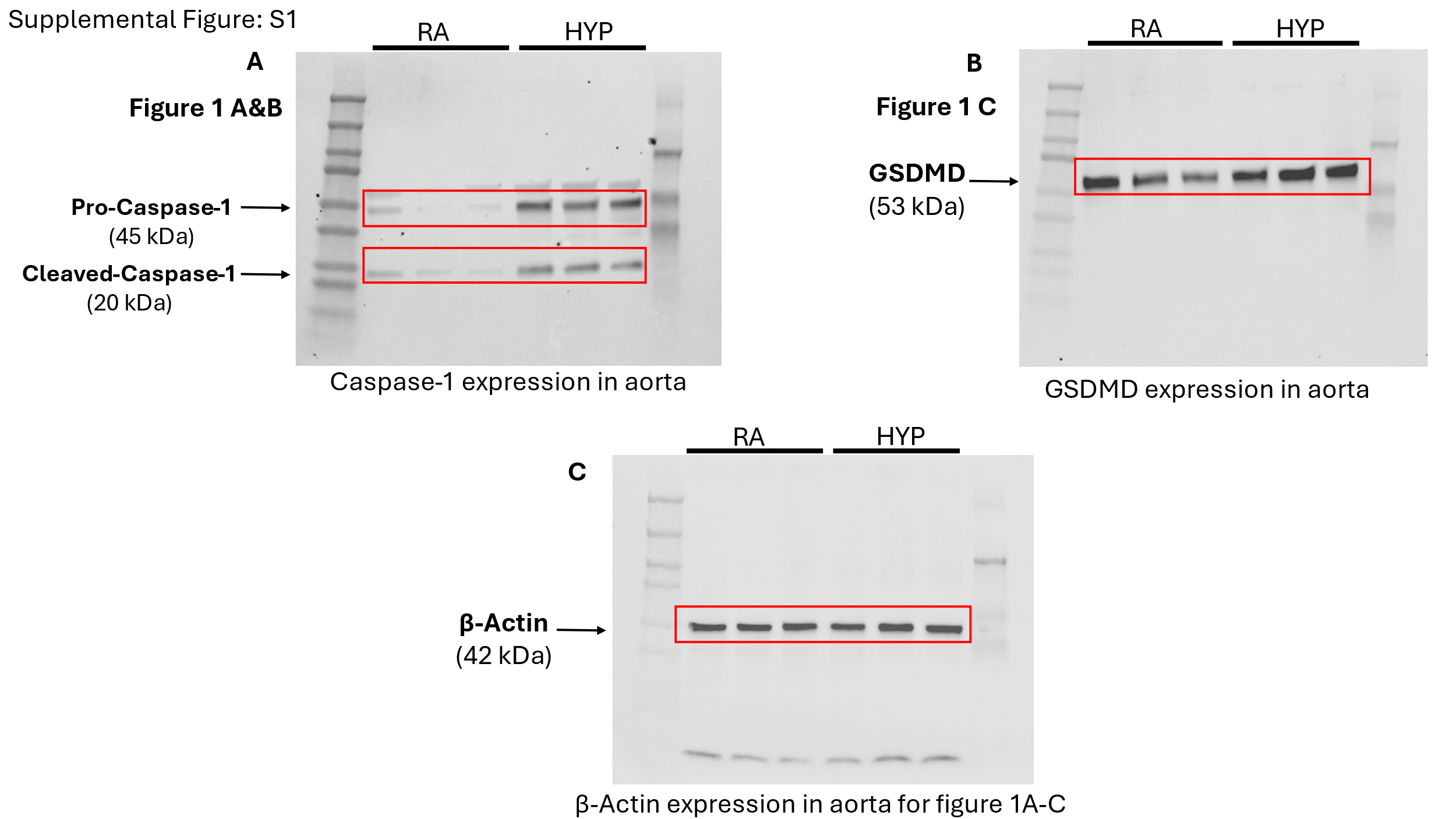


Supplemental figure S1A-C: Original immunoblots for Figure 1 A-C of the manuscript showing Pro-caspase-1, cleaved caspase-1, Gasdermin (GSDMD) and β-Actin expression in the aorta; RA=room air; HYP=hyperoxia.


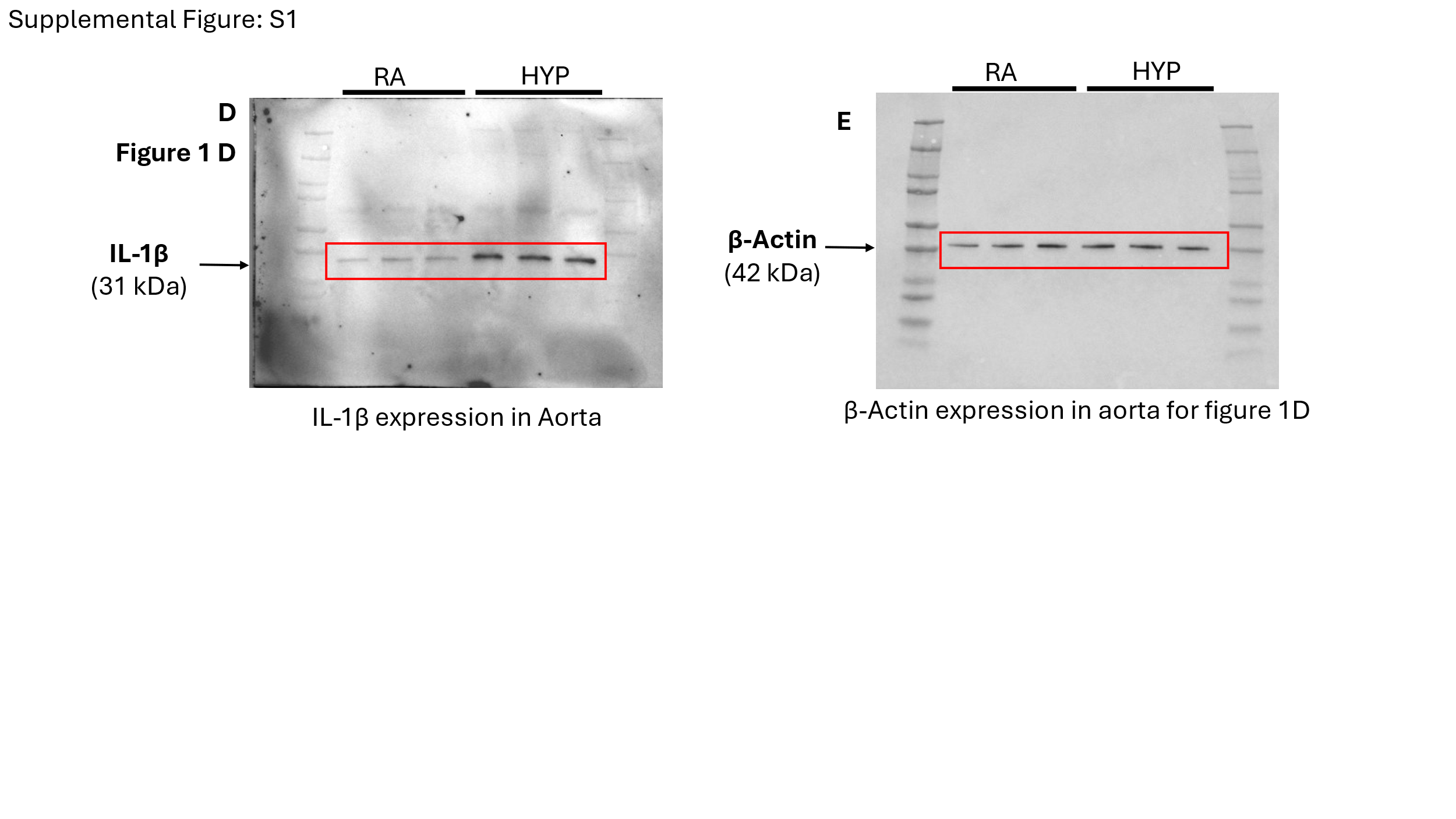


Supplemental figure S1D-E: Original immunoblots for Figure 1 D of the manuscript showing TGF-β1 and β-Actin expression in the aorta; RA=room air; HYP=hyperoxia.


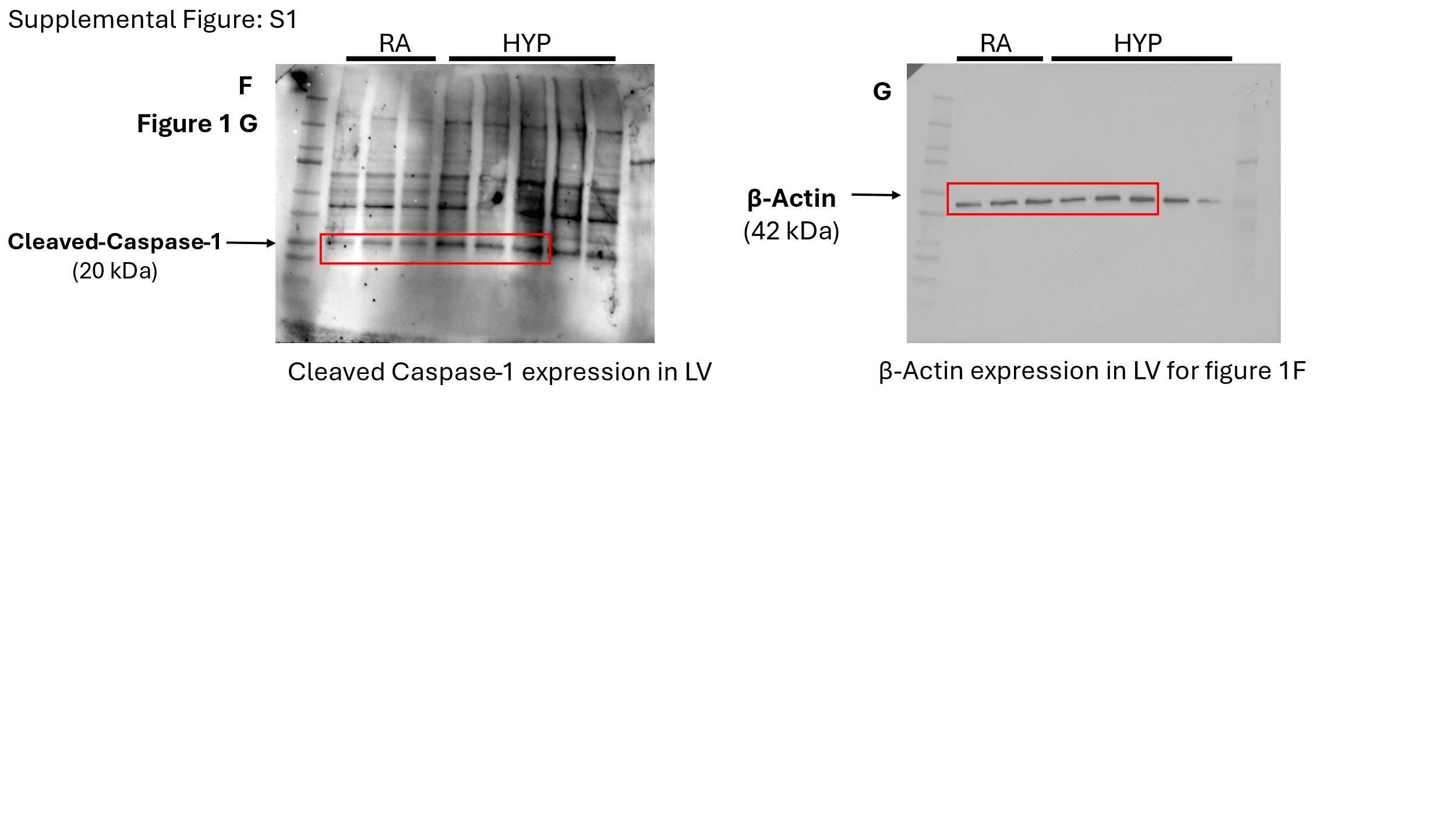


Supplemental figure S1F-G: Original immunoblots for Figure 1G of the manuscript showing cleaved-caspase-1 and β-Actin expression in the aorta; RA=room air; HYP=hyperoxia.


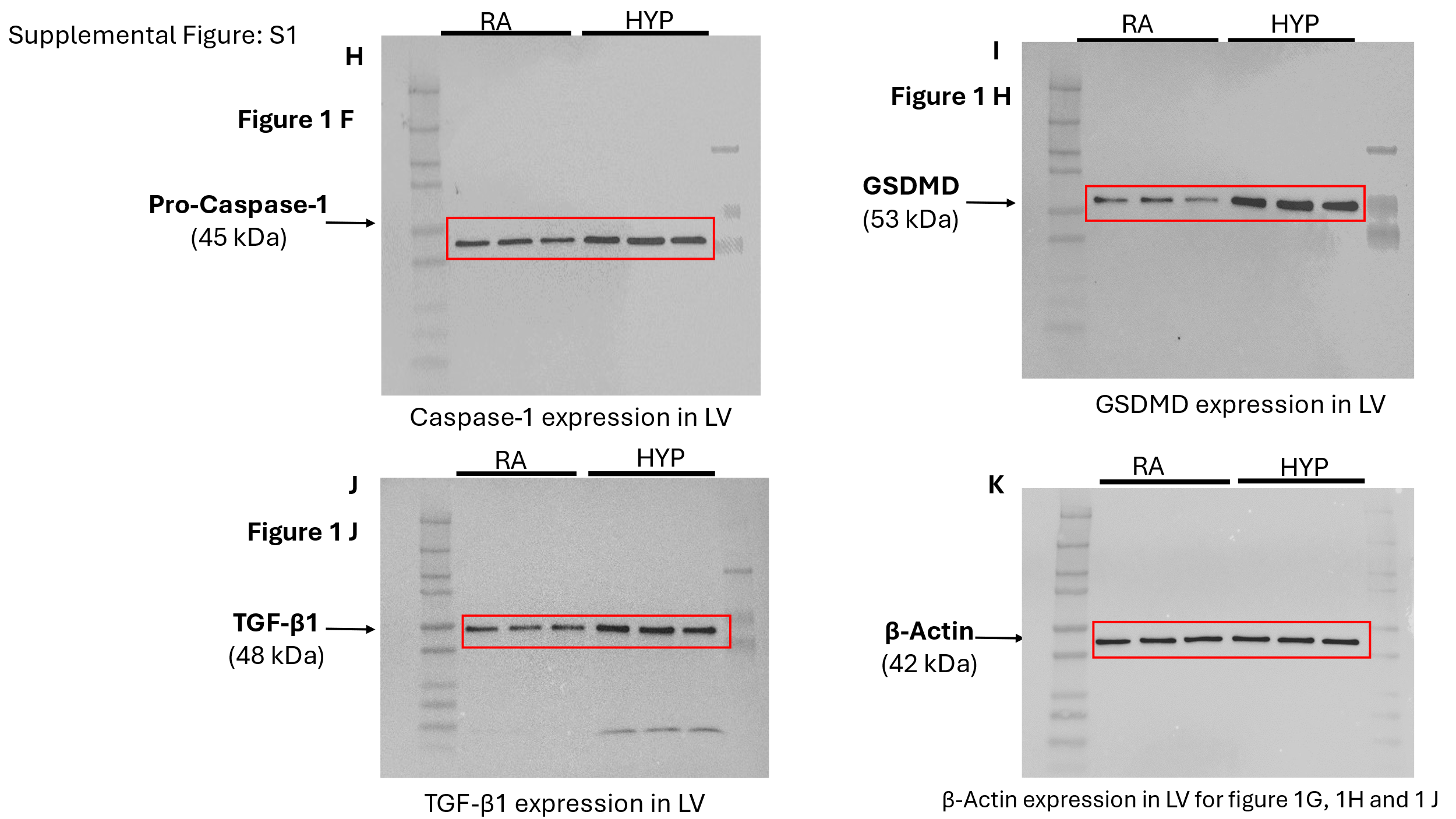


Supplemental figure S1H-K: Original immunoblots for Figure 1F, 1H and 1J of the manuscript showing Pro-caspase-1, Gasdermin (GSDMD), TGF-β1 and β-Actin expression in the aorta; RA=room air; HYP=hyperoxia.


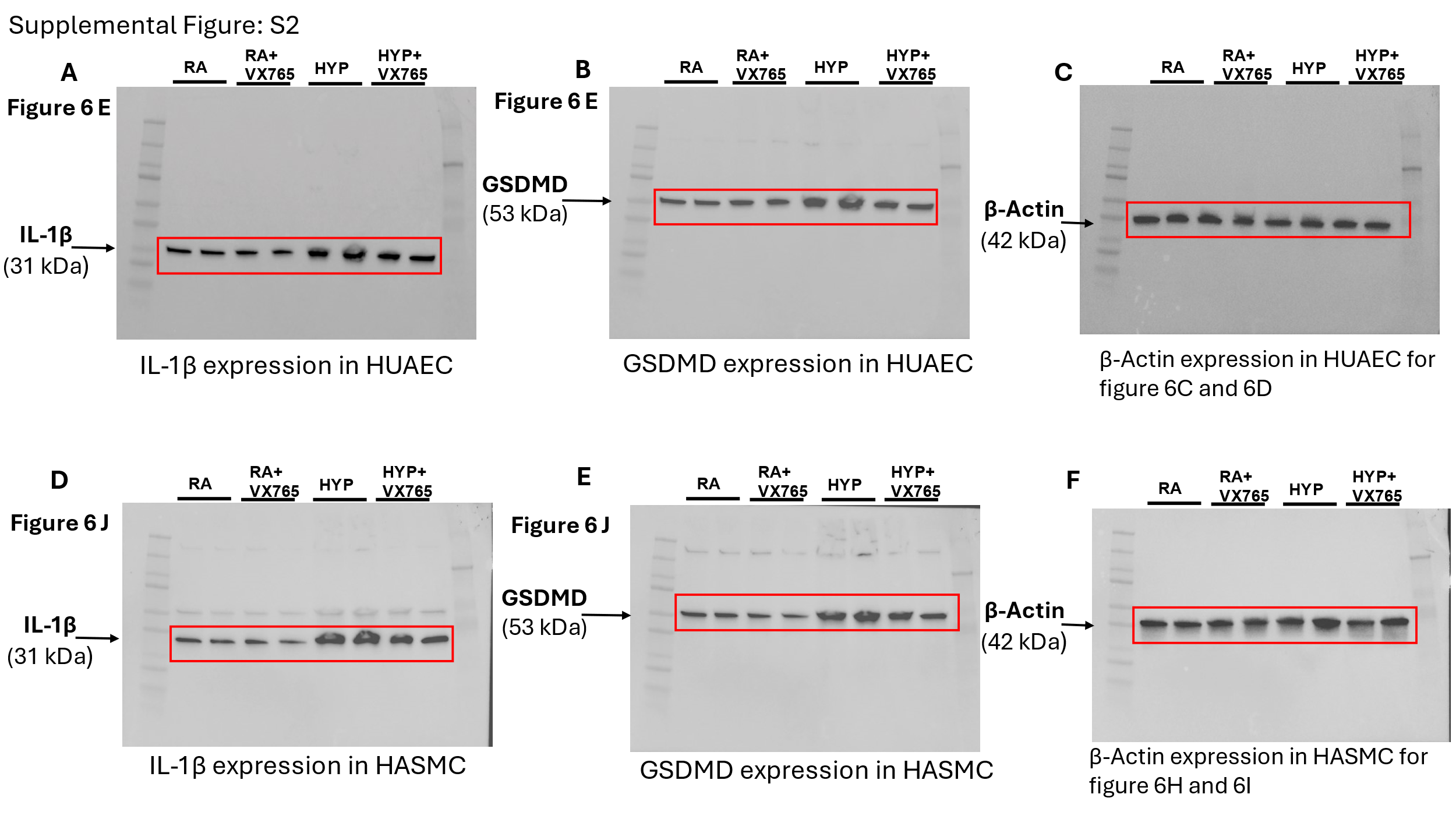


Supplemental figure S2A-F: Original immunoblots for Figure 6E of the manuscript showing IL-1B, Gasdermin (GSDMD), and β-Actin expression in (**A-C)** HUAEC and **(D-F)** HASMC; RA=room air, HYP=hyperoxia, PL=placebo, VX-765=caspase-1 Inhibitor.
